# Sequencing of organellar genomes of *Gymnomitrion concinnatum* (Jungermanniales) revealed the first exception in the structure and gene order of evolutionary stable liverworts mitogenomes

**DOI:** 10.1186/s12870-018-1558-0

**Published:** 2018-12-03

**Authors:** Kamil Myszczyński, Piotr Górski, Monika Ślipiko, Jakub Sawicki

**Affiliations:** 1 Department of Botany and Nature Protection, Faculty of Biology and Biotechnology, University of Warmia and Mazury in Olsztyn, Olsztyn, Poland; 2 Department of Botany, Poznań University of Life Sciences, Poznań, Poland

**Keywords:** Genome rearrangement, Gene order, Liverworts, Plastid genome, Mitochondrial genome, *Marchantiophyta*

## Abstract

**Background:**

Comparative analyses of chloroplast and mitochondrial genomes have shown that organelle genomes in bryophytes evolve slowly. However, in contrast to seed plants, the organellar genomes are yet poorly explored in bryophytes, especially among liverworts. Discovering another organellar genomes of liverwort species by sequencing provides new conclusions on evolution of bryophytes.

**Results:**

In this work, the organellar genomes of *Gymnomitrion concinnatum* liverwort were sequenced, assembled and annotated for the first time. The chloroplast genome displays, typical for most plants, quadripartite structure containing large single copy region (81,701 bp), two inverted repeat regions (8704 bp each) and small single copy region (20,179 bp). The gene order and content of chloroplast are very similar to other liverworts with minor differences observed. A total number of 739 and 222 RNA editing sites were predicted in chloroplast and mitochondrial genes of *G. concinnatum*. The mitochondrial genome gene content is also in accordance with liverworts except few alterations such as: intron loss in *cox1* and *atp1* genes. Nonetheless the analysis revealed that *G. concinnatum* mitogenome structure and gene order are rearranged in comparison with other mitogenomes of liverworts. The causes underlying such mitogenomic rearrangement were investigated and the probable model of recombination was proposed.

**Conclusions:**

This study provide the overview of mitochondrial and chloroplast genome structure and gene order diversity of *Gymnomitrion concinnatum* against the background of known organellar genomes of liverworts. The obtained results cast doubt on the idea that mitogenome structure of early land plants is highly conserved as previous studies suggested. In fact is the very first case of recombination within, evolutionary stable, mitogenomes of liverworts.

**Electronic supplementary material:**

The online version of this article (10.1186/s12870-018-1558-0) contains supplementary material, which is available to authorized users.

## Background

Organellar genomes are widely used as a source of genetic information in evolutionary studies, mainly due to the haploid character and the presence in hundreds to thousands of copies in each cell [1, 2]. In the majority of known organisms the mitogenomes and plastomes are maternally inherited, resulting in presence only single haplotypes of theses genomes in the organism. Several studies described heteroplasmy of plastid genomes [3], however, the most of the studies did not reveal intraindividual polymorphism [4, 5] supporting organellar genomes as a resources for evolutionary studies.

The sequences of complete mitochondrial genomes are mainly used in phylogenetics, phylogeography and population genetics of animals and fungi [6–8], while in plants sciences plastid genomes are mainly used for these purposes.

Compared to the seed plants, the organellar and especially plastid genomes are poorly explored in bryophytes. Up to the date only 15 plastid and 48 mitochondrial complete genome sequences are known for bryophytes genera. Moreover, most of sequenced genomes belongs to just four moss families Funariaceae [9], Grimmiaceae [10, 11], Orthotrichaceae [12–15] and Sphagnaceae [16].

The mitochondrial genomes of early land plants are known from their stable structure in comparison to the seed plants [12, 17]. The liverworts are the oldest evolutionary lineage of sporophytic plants and the most genetically diverse. However, despite high nucleotide variation at inter- and intrageneric level the gene content and order remain almost unchanged since the deepest nodes of liverworts diversification [18–20]. The only observed changes were the intron losses of *atp*1 and *cox*1 genes in the leafy liverworts group [20] and pseudogenization of the nad7 gene in the majority of the liverworts except of *Treubia lacunosa* [18].

This stability seems to be associated with the lack of repetitive sequence in the mitogenomes of early land plants, which are common in seed plants [12]. However, the mitogenomes of the liverworts are poorly explored, even in comparison to the mosses, where up-to-date complete mitochondrial genomes sequences of 6 genera are known.

The available data is even more scarce in case of chloroplast genomes, limited to the genera *Marchantia*, *Pellia*, *Aneura* and *Ptilidium*.

The liverwort *Gymnomitrion concinnatum* (Lightf.) Corda belongs to the family Gymnomitriaceae H. Klinggr. This group includes ten genera (*Acrolophozia*, *Apomarsupella*, *Gymnomitrion*, *Herzogobryum*, *Marsupella*, *Nanomarsupella*, *Nothogymnomitrion*, *Paramomitrion*, *Poeltia*, and *Prasanthus*), the most numerous of which are *Gymnomitrion* (27 species) and *Marsupella* (26 species) [21]. Historically, only two widespread genera (*Gymnomitrion* and *Marsupella*) were considered part of Gymnomitriaceae. Based on the circumscription of the genus *Gymnomitrion* presented by Váňa et al. [21], there are seven species recorded in Poland and Slovakia. Most of them grow in the Tatra Mountains (Western Carpathians). *Gymnomitrion concinnatum* is acidophilus, epilithic and epigeic liverwort that grows on magma (granite) and metamorphic (mostly gneissic) rocks and crystalline slates. Most often it occurs on shelves and crevices of rock walls, less often in loose alpine (and subnival) grasslands and snow-beds with predominance of bryophytes [22]. From Central Europe, phytocoenoses with high occurrence of *G. concinnatum* were described as *Gymnomitrietum concinnati* Herzog 1943 *ex* Philippi 1956 (class: *Grimmietea alpestris* Hadač *et* Vondráček *in* Ježek *et* Vondráček 1962) (compare [22–25]). The known genetic resources of the Gymnomitriaceae are limited to the sequences of ITS and three chloroplast loci [26, 27] of the genera *Gymnomitrion*, *Herzogobryum*, *Marsupella* and *Prasanthus*.

In the present study we sequenced, assembled, annotated and analysed organellar genomes of *Gymnomitrion concinnatum*, which provide new insights into evolution of mitogenomes and plastomes in liverworts.

## Results & discussion

### The characteristics of chloroplast genome of *Gymnomitrion concinnatum*

**Fig. 1:**
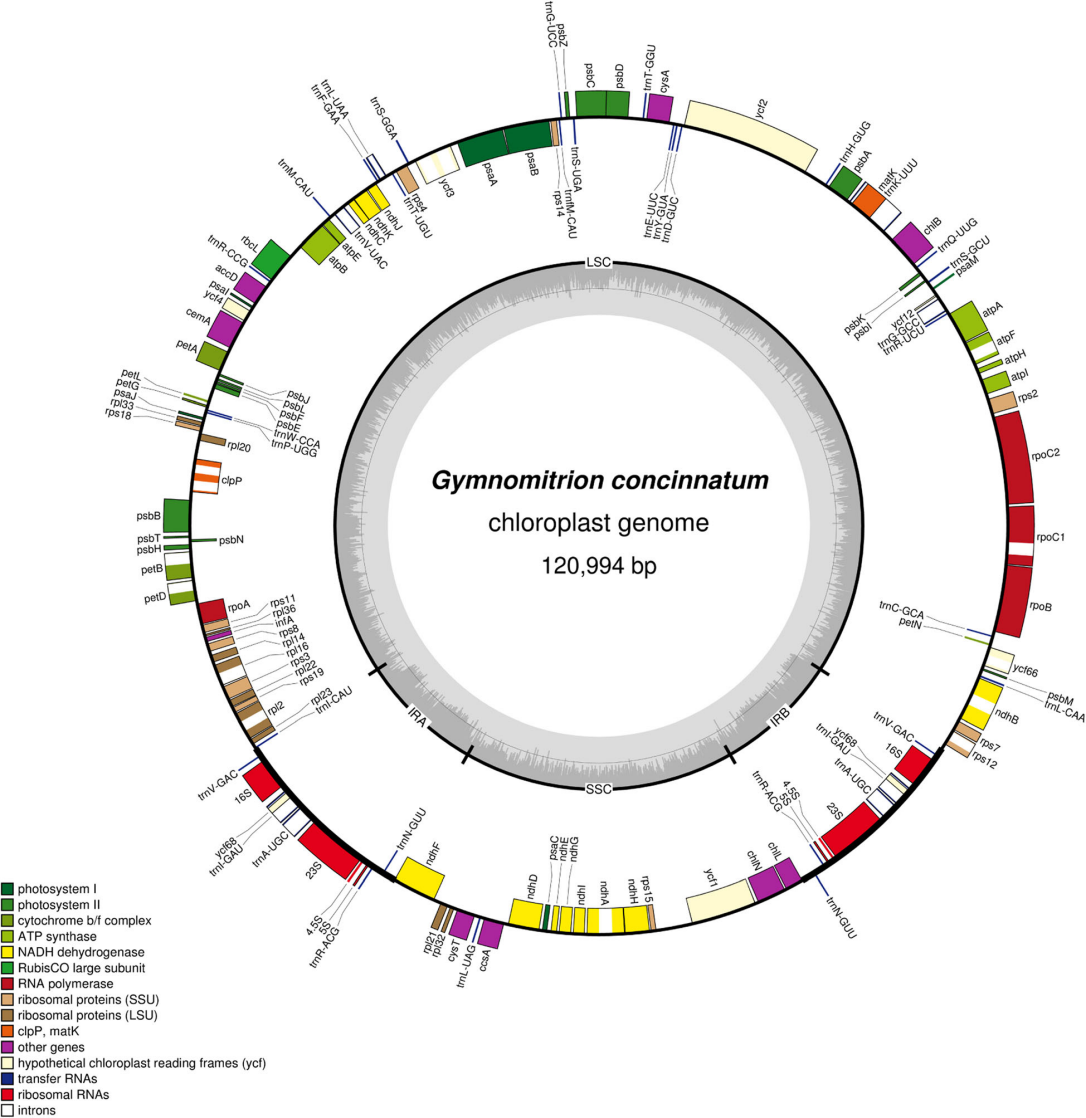
Gene map of the chloroplast of *Gymnomitrion concinnatum*. Genes inside and outside the outer circle are transcribed in counterclockwise and clockwise directions, respectively. The genes are color-coded based on their function. The inner circle visualizes the G/C content

The plastome of *Gymnomitrion concinnatum* is 120,994 bp long with a structure typical for most plants, including a pair of IR regions (each of 8704 bp) separated by LSC (81,701 bp long) and SSC (20,179 bp) regions (Fig. 1). The plastome is almost 2000 bp longer than the second longest known leafy liverwort plastid genome of *Ptilidium pulcherrimum*, however length seems to be variable at the genus level. Comparative analysis of the chloroplast genomes of six *Aneura pinguis* cryptic species revealed that the length of the chloroplast genomes ranged from 120,698 to 121,140 bp [19]. The first sequenced liverworts plastome of *Marchantia paleacea* [28] and later sequenced of the same species differ in length by 390 bp. The changes in length of the plastome could have evolutionary significance, however with limited availability of liverworts plastome sequences, it is too early to conclude about possible variation of plastome size in the leafy liverworts lineage.

The GC content of the *G. concinnatum* plastome is 34.5% and falls within the range of other known liverworts, were GC content ranges from 26.6% in *Marchantia polymorpha* to 40.6% in *Aneura mirabilis* [29], but was higher than in *Ptilidium pulcherrimum* (33.2%), the first leafy liverwort for which the complete plastid genome was sequenced [30].

As in the closest known relative with a plastome sequence, *P. pulcherrimum*, the plastome of *Gymnomitrion* consist of 121 unique genes, including 81 protein-coding genes, 6 genes of unknown function (*ycf* genes), 4 ribosomal RNAs and 30 transfer RNAs (Additional file 1: Table S2). The gene order and content seems to be stable in leafy (*Gymnomitrion concinnatum*, *Ptilidium pulcherrimum*) and simple thalloid liverworts (*Aneura pinguis*, *Pellia endiviifolia*). Complex thalloid liverworts (*Marchantia paleacea, M. polymorpha*) have two more genes: *cys*A and *cys*T. Heterotrophic *Aneura mirabilis* has a reduced plastome due to lost or pseudogenization of genes involved in the process of photosynthesis [29].

Besides slight differences the comparative analysis of two known leafy liverworts plastomes revealed similarity in size and functionality of genes. The plastome of *Gymnomitrion concinnatum* is 1987 bp longer than *Ptilidium pulcherrimum* mainly due to over 500 bp insertion between *trn*H-GUC and *ycf*2 genes. The second insertion is located within the mentioned *ycf*2 gene, which is 598 bp longer in *Gymnomitrion* than in *Ptilidium* (5828 bp vs 5242 bp).

### Features of *Gymnomitrion concinnatum* mitochondrial genome

**Fig. 2:**
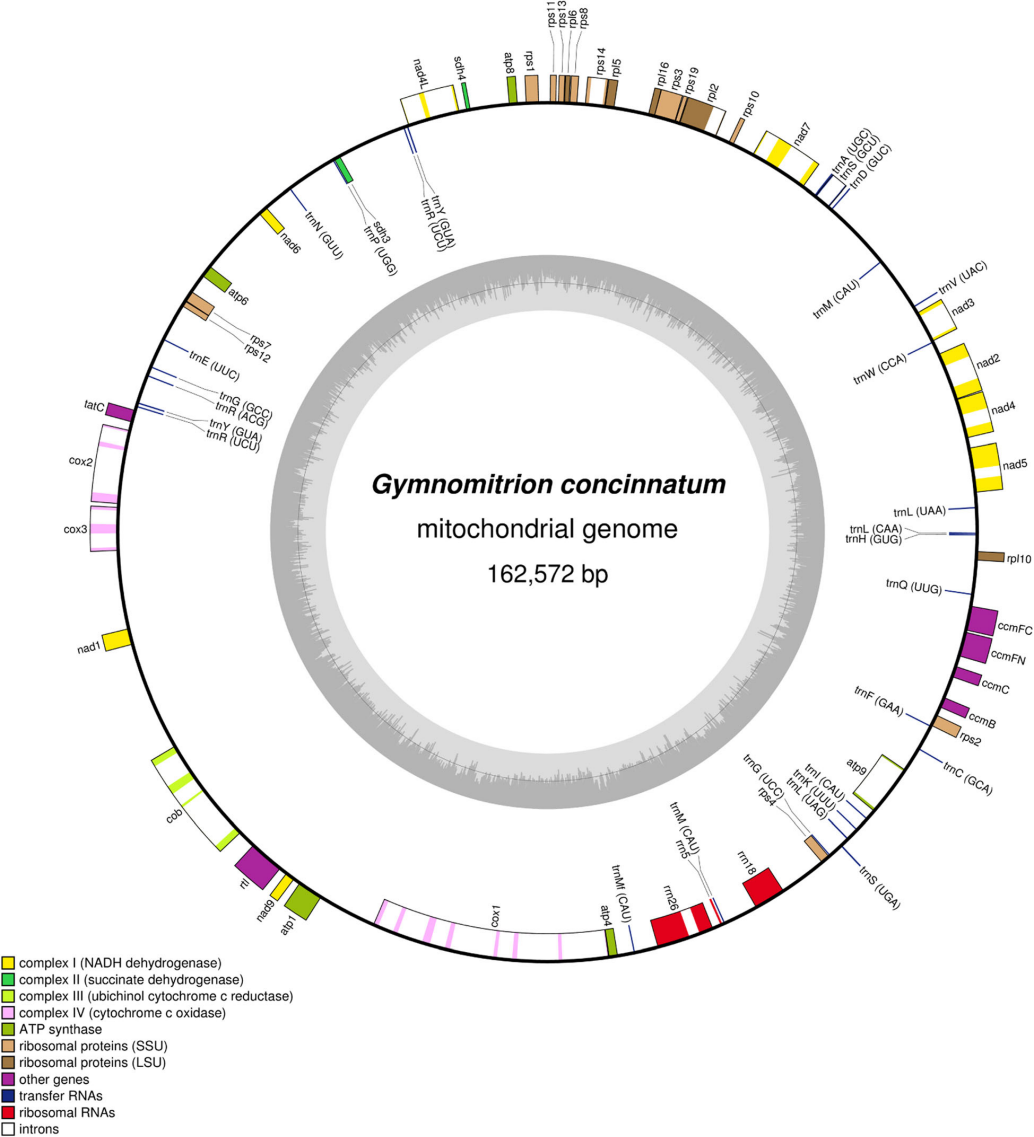
Gene map of the mitochondrion of *Gymnomitrion concinnatum*. Genes inside and outside the outer circle are transcribed in counterclockwise and clockwise directions, respectively. The genes are color-coded based on their function. The inner circle visualizes the G/C content

**Fig. 3:**
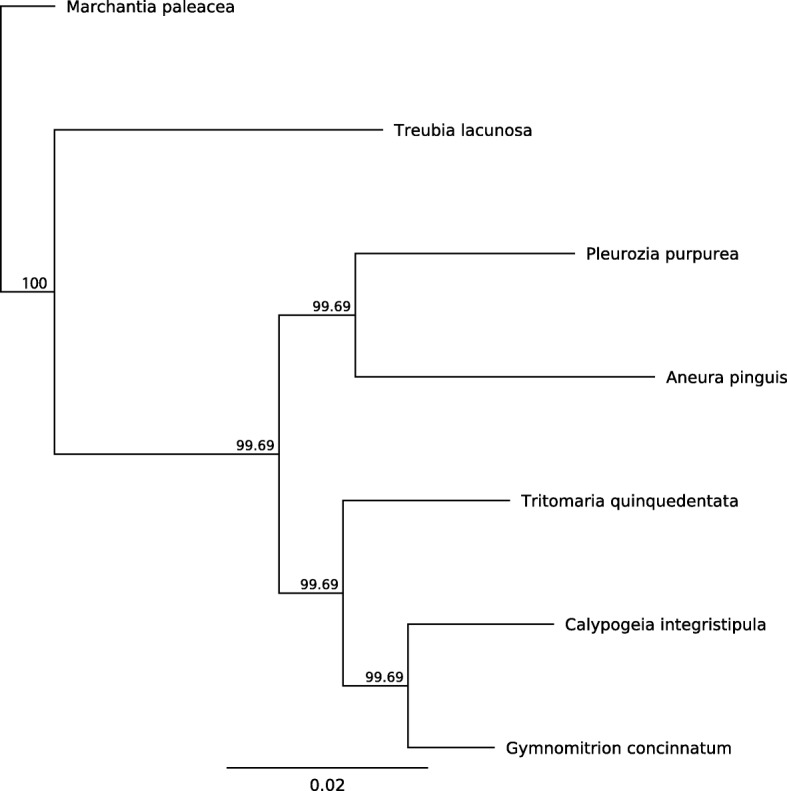
Phylogenetic relationships among *Gymnomitrion concinnatum* and other liverworts based on whole set of protein-coding sequences of mitochondrial genomes. The posterior probability values are given at the nodes

The complete mitochondrial genome of *G. concinnatum* is 162,574 bp in length (Fig. 2), which is in accordance with other reported Marchantiophyta mitogenomes, i.e. *Aneura pinguis* [19], four *Calypogeia* species [20], *Marchantia paleacea* [31], *Pleurozia purpurea* [17], *Treubia lacunosa* [18], *Tritomaria quinquedentata* [20]. The length of aforementioned mitochondrial genomes ranges from 142,510 (*T. quinquedentata*) to 186,609 bp (*M. paleacea*). Overall GC content of the mtDNA is 44.7%, which is similar to other known liverworts mitochondrial genomes (42.2–47.4%). The mitogenome of *G. concinnatum* contains 70 unique genes, including 42 protein-coding genes, 3 ribosomal RNAs and 25 transfer RNAs (Additional file 1: Table S2), which is a typical set of mitochondrial protein-coding genes involved in respiration and protein synthesis. The phylogenetic tree constructed on the basis of 38 protein-coding sequences of mitogenomic sequences of seven liverworts is in accordance with Marchantiophyta clade phylogeny [32] (Fig. 3).

The *nad7* protein-coding sequence was identified in *G. concinnatum* mitogenome as a pseudogene. The absence of *nad*7 CDS is not surprising, in view of the fact that previously conducted analyses on liverworts have shown that *nad*7 occurs as pseudogene in Marchantiopsida and Jungermanniopsida [17, 19, 20, 31, 33]. The only liverworts that preserved functional *nad7* belong to Haplomitriopsida clade: *Treubia lacunosa* and *Haplomitrium mnioides* [18, 33]. Interestingly, the ORF of the first exon in *G. concinnatum nad*7 was preserved and showed 84.8 and 82.8% sequence identity to *T. lacunosa* and *H. mnioides*, respectively. None of other known liverworts mitochondrial genomes preserved ORF of the first exon of *nad7* gene [17, 19, 20, 31, 33].

Other gene structure alterations observed in *G. concinnatum* mitogenome were intron losses in *cox*1 and *atp*1 genes. *G. concinnatum cox*1 contained 7 introns while other liverworts, reported earlier [17–19, 31], contain 9 introns (Additional file 2: Figure S1). *cox*1 intron loss has been recently reported in another Jungermanniales - *Tritomaria quinquedentata* and *Calypogeia* species (loss of 4 and 3 introns, respectively) [20]. All of these observations suggest that intron loss in *cox*1 is a feature specific to Jungermanniales species. Additionally, the *atp*1 gene lost both introns in *G. concinnatum* mitochondrial genome and is intronless. This phenomenon was also reported in previous studies on *Calypogeia* species, as well as *Tritomaria quinquedentata* and *Treubia lacunosa* species [18, 20].

### Prediction of RNA editing sites of chloroplast genes

RNA editing is the process that alters the identity of nucleotides in RNA sequence or that add or delete nucleotides so that the mature RNA sequence differs from that defined in the genome [34–36]. In order to analyse possible RNA post-transcriptional modifications in protein-coding sequences, 87 plastid CDSs were investigated using PREPACT 2.0 [37]. A total number of 739 RNA editing sites were predicted in chloroplast genes of *G. concinnatum*. The C to U substitutions accounted for 60.1% (444 substitutions), while U to C substitutions accounted for 39.9% (295) of total RNA editing sites. Three substitutions affected ORF of two CDSs: *ccsA* and *petD*, where glutamine codon were altered to stop codon, as well as *atpF*, where threonine codon was altered to methionine codon which resulted in restoration of ORF of the gene. The substitution in *atpF* seems to be crucial for providing protein product. Considering aforementioned substitutions, the CDSs of the two genes are consistent with other liverworts. The highest RNA editing site content was observed in *petL* gene (4.2% of the CDS nucleotides were altered), however the coding sequence of the gene is only 96 bp length. It is worth mentioning that in 8 of 15 CDSs of subunits of photosystem II no RNA editing sites were found (Additional file 3: Table S3).

### Prediction of RNA editing sites of mitochondrial genes

A total number of 222 RNA editing sites were predicted in 42 CDSs of *G. concinnatum* mitogenome. The C to U substitutions accounted for 76.6% (170 substitutions), while U to C substitutions accounted for 24.4% (52) of total RNA editing sites. The plastome CDSs (739 substitutions while 71,379 bp total length) contained 1.6 times as many RNA editing sites as mitogenome CDSs (222 substitutions while 33,534 bp total length). Two substitutions identified within mitogenome altered threonine codon to methionine codon which result in occurrence of start codons (*nad2* and *nad6* genes) and one substitution altered glutamine codon to stop codon (*atp9* gene). Despite the aforementioned three substitutions the predicted translation products of the CDSs are consistent with corresponding CDSs of other liverworts. In fact due to RNA editing modifications the proper ORF of *nad2* and *nad6* genes are restored. The highest RNA editing site content was observed in *ccmFC* gene sequence (2.3% of the CDS nucleotides were altered) as well as the average RNA editing site content was the highest among cytochrome c maturation coding genes (1.5%). On the other hand, no substitutions were found in six CDSs: *cox2*, *nad1*, *rpl6*, *rpl16*, *rps4*, *rps13* and *rps19* (Additional file 4: Table S4).

### Gene order and repeat sequences

**Fig. 4:**
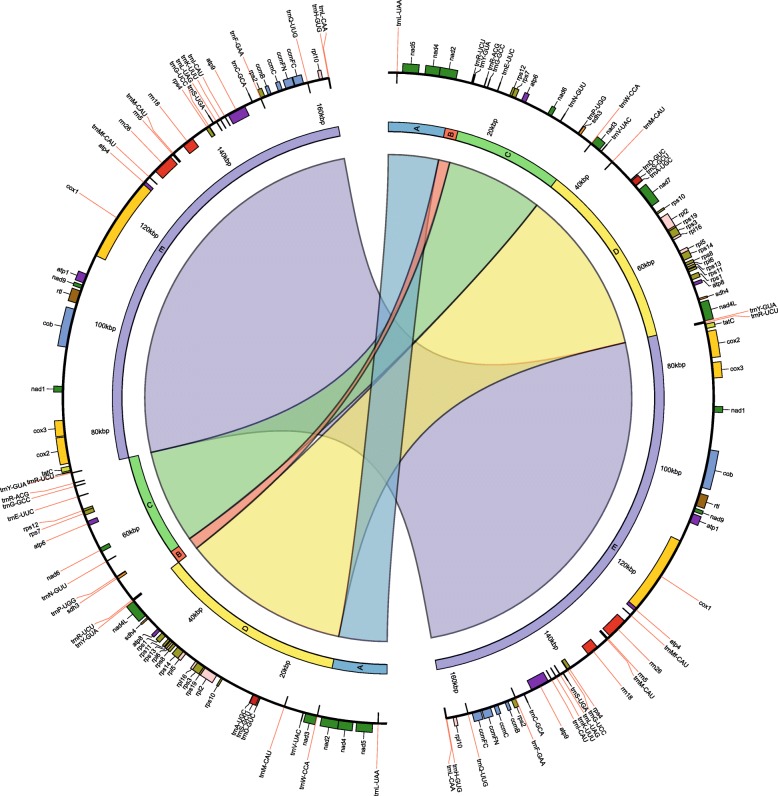
Comparison of *G. concinnatum* (left) and *C. integristipula* (right) mitochondrial genome structure and gene order. The outer track visualizes the genes, tRNA and rRNA order of both mitogenomes. The inner track visualizes rearrangements of *G. concinnatum* mitogenome and its regions (as LCBs) relatively to *C. integristipula* mitogenome as representative of other liverworts. The coloured links between LCBs represent location of the same regions. The strandedness of each region is also preserved: outer blocks represent the forward strand, while inner blocks the reverse strand

Liverworts are considered to be conservative in terms of mitochondrial gene order evolution [12, 17, 18]. All complete liverwort mitochondrial genome sequences published have shown that the gene order is preserved in Marchantiophyta. These analyses included six species of liverworts from the orders Treubiales, Marchantiales, Pleuroziales, Metzgeriales and Jungermanniales, spanning the Marchantiophyta [17–20, 31]. Therefore *Gymnomitrion concinnatum* was expected to preserve the same mitochondrial gene order. In order to verify structural homology of *G. concinnatum* mitogenome with known species of liverworts, the mitogenome sequences of *G. concinnatum* and *C. integristipula* were aligned using MAUVE software [38] and then visualized as genome maps comparison using Circos [39]. The analysis revealed that *G. concinnatum* mitogenome structure is rearranged in comparison with other Marchantiophyta mitogenomes. In comparison with *C. integrisipula*, five locally collinear blocks (LCB) were identified among these two mitochondrial genomes (Fig. 4). First and last LCB (A and E) were located within the same regions on both mitogenomes. However middle three LCBs of *G. concinnatum* (B, C and D) were arranged in a different order. Two of these three LCBs were inverted in relation to *C. integristipula* mitogenome. All LCBs started and ended within intergenic spacers. The mitogenome structure was in accordance with mapping to reference and de novo assembly approaches with high coverage values observed at LCB junctions. Additionally, the aforementioned structure of mitogenome was independently confirmed with the use of PCR method (Additional file 5: Figure S2). The above observations cast doubt on the idea that mitogenome structure of liverworts or even whole Bryophyte is highly conserved as suggested in previous studies [12, 17, 18].

Rearrangements of mitochondrial genome structure of seed plant are usually connected with occurrence of repeated sequences [40–42]. Repeats of different sizes have been observed in higher plants mitogenomes: large (> 500 bp), intermediate (50–500 bp) and small (< 50 bp). Large and intermediate repeats are considered to be involved in recombination of mitochondrial genomes structure [41, 43]. Such repeated sequences can pair up, recombine and form different configuration of mitogenome as a result [17]. Considering the above and the fact that mitogenome structure rearrangements among Marchantiophyta and Bryophyta have not been reported previously, further investigation on sequence repeats and recombination was undertaken in this study.

Consequently, repeated sequences, exceeding 100 bp in size, were identified in *G. concinnatum* mitogenome sequence. This analysis distinguished ten pairs of sequence repeats, varying from 107 bp to 566 bp length, which overall account for 3.1% of mitochondrial genome sequence. It turned out that the two longest repeats, 566 bp (97.7% sequence identity) and 435 bp length (95.9% sequence identity) were located on junctions between aforementioned LCBs. Therefore, the two repeated sequences of first pair (R1) were located on the edges of B-C and D-E LCBs, while the two repeated sequences of second pair (R2) were located on the edges of A-B and D-C LCBs (Fig. 4).

**Fig. 5:**
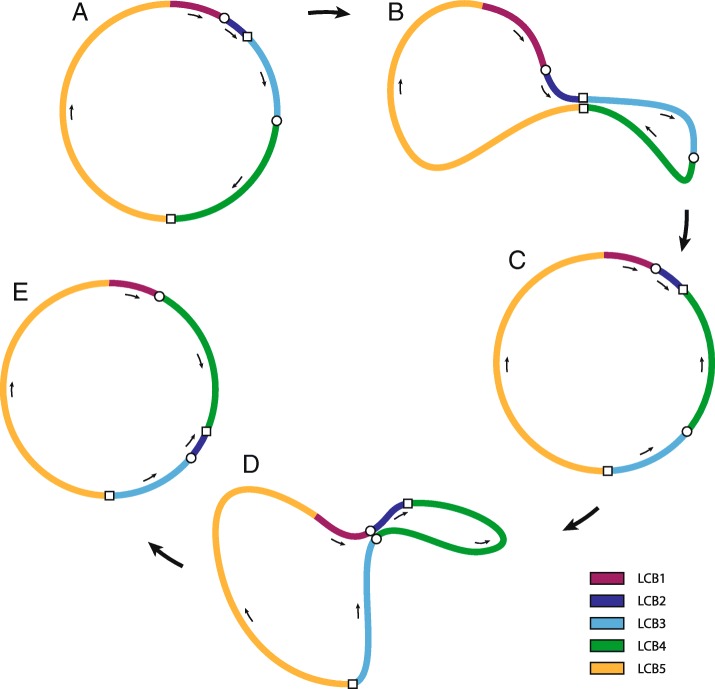
Probable recombination model that occurred in mitochondrial genome of *Gymnomitrion concinnatum*. First stage (**a**) represents the mitochondrial structure typical for the rest of known mitogenomes of liverworts while the last stage (**e**) represents mitochondrial structure that was identified in *G. concinnatum*. Rearrangement within R1 repeats (**b**) results with intermediate configuration of mitogenome (**c**). Second rearrangement (**d**), within R2 repeats, finally leads to  configuration of *G. concinnatum* mitogenome. The colored lines represent different LCBs. The two pairs of repeated sequences are depicted (pair of white squares and pair of white circles)

Considering unusual configuration of *G. concinnatum* mitogenome and location of repeated sequences, the model of recombination was proposed (Fig. 5). It is likely that two rearrangements within mitogenome have occurred one after the other. First, starting with LCBs arranged in order common for liverworts i.e. A, B, C, D, E, the rearrangement within R1 repeats has taken place resulting with following order of LCBs: A, B, D (inverted), C (inverted) and E. Next, the second rearrangement within R2 repeats has occurred resulting with following order of LCBs: A, D, B (inverted), C (inverted) and E, which is mitochondrial genome configuration identified in *G. concinnatum* (Fig. 4).

**Fig. 6:**
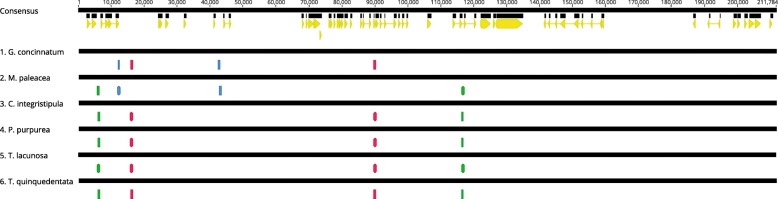
Location of repeated sequences within alignment of six mitogenomes of liverworts. Three pairs of repeated sequences were identified: R1 (blue blocks), R2 (pink blocks) and R3 (green blocks). The yellow blocks depict coding-sequences of genes on the consensus sequence. The mitochondrial genome of *G. concinnatum* have been artificially rearranged in order to obtain the same mitogenome structure as in other species

In order to investigate if the same pattern of repeated sequences occurs within mitochondrial genomes of other species of the same division, the analyses of sequence repeats among 6 complete mitogenome sequences of aforementioned liverworts were conducted. In every mitochondrial genome sequence, except *A. pinguis,* two pairs of repeated sequences exceeding 400 bp and 90% of identity were found. Considering the location of repeated sequences on mitogenomes, three different pairs were distinguished (Fig. 6). Four of six species contained the same pattern of repeated sequences: R2 and R3 repeats, where R2 repeats were located within *nad2*-*rps12* and *nad4L-tatC* intergenic regions while R3 repeats were located within *nad5-nad4* intergenic spacer and the second intron of *cob*. Interestingly, *M. paleacea* mitogenome contained only one of aforementioned repeated sequences pairs - R3, but also R1 - identified in *G. concinnatum* mitochondrial sequence. It seems that mitochondrial genome of *G. concinnatum* share one pair (R2) identified in *C. integristipula*, *P. purpurea*, *T. quinquedentata* and *T. lacunosa* and another pair (R1) identified in *M. paleacea*. However further analysis revealed that R3 pair, absent in *G. concinnatum* mitogenome, was not completely missing in that genome. The second mate of R3 repeated sequence pair located within second intron of *cob* gene was still present while deletion exceeding 600 bp within *nad5-nad4* intergenic spacer caused disappearance of the first mate of R3 pair.

Considering the unique pattern of repeated sequences of *G. concinnatum* mitogenome, it seems plausible that described patterns of repeated sequences might be crucial for maintenance of gene order and the variations of specific patterns may cause recombination of mitochondrial genome and therefore gene order rearrangement. Up to now variations of relative placement of the genes within mitochondrial genomes caused by homologous recombination were identified only among seed plants. The mitochondrial gene order rearrangements have been found on different levels of phylogenetic classification of these plants [8, 41, 44]. The results obtained here provide the first evidence of rearrangements in the structure and gene order of widely considered as evolutionary stable mitochondrial genomes of liverworts.

## Conclusions

The results obtained in this study provide the overview of mitochondrial and chloroplast genome structure and gene order of *Gymnomitrion concinnatum* against the background of known organellar genomes of liverworts. The complete organellar genome sequences of *G. concinnatum* were fully sequenced for the first time extending the knowledge of the poorly explored organellar genomes of bryophytes. Almost all aspects of organellar genomes evolution such as diversity in gene content, genome size and sequence similarity, seems to be rather conservative in *G. concinnatum* and highly similar to other liverworts.

Nonetheless the most relevant finding of the study, in contrast, is the discovery of rearrangements in the structure and gene order of *G. concinnatum* mitochondrial genome. It is the first case of mitochondrial recombination among liverworts. The recombination activity of plant mitochondria plays a major role in the evolution of mitochondrial genomes [45]. Previous studies have concluded that the mitogenome of liverworts exhibits conservative evolution contrary to highly dynamic evolution in seed plants [17]. The findings provided by this study strongly support the hypothesis that gene order rearrangements of mitochondrial genome structure are not just limited to seed plants which is in opposite to generally accepted statement that the mitogenome gene order of liverworts is constant [12]. Considering the fact that up to now only 7 mitogenomes of liverworts, including *G. concinnatum*, have been fully sequenced it is likely that other mitochondrial genomes of this plant group may be also rearranged in their structure and gene order. However further research providing knowledge on organellar genomes of another species of bryophytes need to be conducted to test above hypothesis.

## Methods

### Plant material

The specimen details were as follow: *Gymnomitrion concinnatum* (Lightf.) Corda, Slovakia, High Tatra Mountains, Výsňé Kôprovické sedlo pass (Liptovské kopy), 49.19040°N, 19.96641°E, alt. 1910 m a.s.l., fine-grained screen with high participation of lichens, leg., det. P. Górski, 4.09.2014. The DNA was extracted using the Zymo Plant/Seed DNA kit (Zymo Research, Irvine, CA, USA). One individual from a one year old herbarium specimen was ground with silica beds in a MiniBead-Beater homogenizer for 50 s and subsequently processed according to the manufacturer protocol. DNA quantity was estimated using Qubit fluorometer and Qubit™ dsDNA BR Assay Kit (Initrogen, Carsbad, NM, USA). DNA quality was checked by electrophoresis in 0.5% agarose gel stained with Euryx Simple Save (Eurx, Gdańsk, Poland).

### Genome sequencing, assembly and annotation

The genomic library was constructed with TruSeq Nano DNA kit (Illumina, San Diego, CA, USA) and was sequenced using HiSeqX (Illumina) to generate 150 bp paired-end reads at Macrogen Inc. (Seoul, Korea) with 350 bp insert size between paired-ends. Afterwards, 20,166,426 sequencing reads were cleaned by removing the adaptor sequences and low-quality reads with Trimmomatic v0.36 [46]. The filtered reads were assembled using SPAdes 3.12.0 [47]. Reference mitogenome sequence of *Calypogeia integristipula* (NC035977.1) and plastome sequence of *Ptilidium pulcherrimum* (NC015402.1) were used to identify organellar genomes of *G. concinnatum* among the generated contigs. To verify de novo assembly iterative mapping was carried out independently for each genome using Geneious R8 software [48]. The chloroplast and mitochondrial genome sequences of *G. concinnatum* had 1440x and 264x coverage depth, respectively.

Genes were identified and annotated based on the closest known organellar genomes of related species to *G. concinnatum* i.e., *Calypogeia integristipula*, *Tritomaria quinquedentata*, *Pleurozia purpurea*, *Ptilidium pulcherrimum* and *Aneura pinguis*. Predictions were made using Geneious R8 software [48] and BLAST+ 2.8.0 tool [49]. Annotated sequences of *G. concinnatum* chloroplast and mitochondrial genome were submitted to GenBank under MH705066 and MH705065 accession number, respectively. Circular genome maps were created using the OGDraw software [50]. To verify gene order of *G. concinnatum* mitogenome the mitogenome sequences of *G. concinnatum* and *C. integristipula* were aligned using Mauve 2.3.1 [38]. The comparative analysis of the two mitogenomes was visualized using Circos plot [39].

### PCR analysis

The junction regions between rearranged LCBs of *G. concinnatum* mitogenome were confirmed using PCR. Four primers pairs were designed based on the nucleotide sequences that overlap edges of four LCBs pairs. The sequences of primers with expected amplicons lengths are given in Additional file 6: Table S1. PCR reactions were performed in 25 μL of a reaction mixture containing 20 ng of DNA, 1x PCR buffer, 1.5 mM MgCl_2_, 200 μM dNTP (dATP, dGTP, dCTP, dTTP), 1.0 μM of each primer and 1 U RUN polymerase (A&A Biotech, Gdańsk, Poland). Reactions were performed under the following thermal conditions: (1) initial denaturation—4 min at a temperature of 94 °C; (2) denaturation—45 s at 94 °C; (3) annealing—50 s at 53 °C for, (4) elongation–60 s at 72 °C; (5) final elongation—7 min at 72 °C. Stages 2–4 were repeated 30 times. PCR products were separated in the QIAxcel capillary electrophoresis system, using the Qiaxcel High Resolution Kit; with the 15–3000 bp alignment marker (Qiagen) and 100–2500 DNA size marker. Standard OM500 settings were used as the electrophoresis program. The size of the obtained amplicons were determined by using BioCalculator software (Qiagen).

### Phylogenomics reconstruction

Mitogenomic sequences of seven liverworts i.e. *Aneura pinguis*, *Calypogeia integristipula*, *Marchantia paleacea*, *Pleurozia purpurea*, *Treubia lacunosa, Tritomaria quinquedentata* available in GenBank and *Gymnomitrion concinnatum* presented in this study, were used for the phylogenetic analysis. First, a set of 38 protein-coding sequences (Additional file 1: Table S2), present in each mitogenomes, were extracted, concatenated and aligned using Geneious R8 [48] and MAFFT [51]. Next, based on the alignment, Bayesian analysis was conducted using MrBayes 3.2.1 [52], including *M. paleacea* as an outgroup. The MCMC algorithm was run for 2,000,000 generations (sampling every 1000) with four incrementally heated chains. The first 1000 trees were discarded as burn-in. The remaining trees were used to generate the consensus tree.

### Prediction of RNA-editing sites

To predict editing sites within protein-coding sequences of 87 chloroplast and 42 mitochondrial genes, PREPACT 2.0 [37] tool was used with 0.001 e-value cutoff.

## Additional files

Additional file 1: **Table S2.** Gene contents in organellar genomes of *Gymnomitrion concinnatum.* (DOC 50 kb)

Additional file 2: **Figure S1.** Structure of *cox1* gene among liverworts. The light grey coloured blocks depict introns while dark grey blocks depict exons of the gene. The *M. paleacea cox1* gene is representative of the rest of liverworts. The consensus graph presents sequence identity among these four sequences (the greener regions the higher identity). (PDF 580 kb)

Additional file 3: **Table S3.** Predicted RNA editing sites within chloroplast genes of *Gymnomitrion concinnatum.* (DOC 149 kb)

Additional file 4: **Table S4.** Predicted RNA editing sites within mitochondrial genes of *Gymnomitrion concinnatum.* (DOC 84 kb)

Additional file 5: **Figure S2.** The PCR validation of the mitogenome structure. Four electropherograms of amplicons obtained as a result of PCR analysis. The x-axis represents time while y-axis represents relative fluorescence. (PDF 209 kb)

Additional file 6: **Table S1.** Sequences of primers used in the present study. (DOC 37 kb)

## Abbreviations

CDS: Coding sequence
IR: Inverted repeat region
LCB: Locally collinear block
LSC: Large single copy region
ORF: Open reading frame
SSC: Small single copy region
