# Supplementary material for: Sequencing of organellar genomes of Gymnomitrion concinnatum (Jungermanniales) revealed the first exception in the structure and gene order of evolutionary stable liverworts mitogenomes
Source: BMC Plant Biol. 2018 Dec 3;18:321. doi: 10.1186/s12870-018-1558-0 (PMC6276189; doi:10.1186/s12870-018-1558-0)
Supplement: Supplementary file 1 — Table S2. Gene contents in organellar genomes of Gymnomitrion concinnatum. (DOC 50 kb) [file 12870_2018_1558_MOESM1_ESM.doc]

Table S2

Gene contents in organellar genomes of *Gymnomitrion concinnatum*

| **Genome** | **Gene group** | **Gene name** |
| --- | --- | --- |
| Chloroplast | Acetyl-CoA subunit | *accD* |
|  | ATP synthase subunits | *atpA*, *atpB*, *atpE*, *atpF*, *atpH*, *atpI* |
|  | Conserved open reading frames | *ycf1, ycf2, ycf12, ycf66* |
|  | C-type cytochrome synthesis gene | *ccsA* |
|  | Cytochrome subunits | *petA, petB, petD, petG, petL, petN* |
|  | DNA-dependent RNA polymerase | *rpoA, rpoB, rpoC1, rpoC2* |
|  | Envelope membrane protein | *cemA* |
|  | Large subunit of ribosome | *rpl2, rpl14, rpl16, rpl20, rpl21, rpl22, rpl23, rpl32, rpl33, rpl36* |
|  | Light-independent protochlorophyllide reductase subunits | *chlB*, *chlL*, *chlN* |
|  | Maturase | *matK* |
|  | NADH oxidoreductase | *ndhA, ndhB, ndhC, ndhD, ndhE, ndhF, ndhG, ndhH, ndhI, ndhJ, ndhK* |
|  | Photosystem I subunits | *psaA, psaB, psaC, psaI, psaJ, psaM, ycf3, ycf4* |
|  | Photosystem II subunits | *psbA, psbB, psbC, psbD, psbE, psbF, psbH, psbI, psbJ, psbK, psbL, psbM, psbN, psbT, psbZ* |
|  | Protease | *clpP* |
|  | Ribosomal RNA genes | *rrn4.5, rrn5, rrn16, rrn23* |
|  | Rubisco large subunit | *rbcL* |
|  | Small subunit of ribosome | *rps2, rps3, rps4, rps7, rps8, rps11, rps12, rps14, rps15, rps18, rps19* |
|  | Sulphate ABC transporter subunits | *cysA*, *cysT* |
|  | Transfer RNA genes | *trnA-UGC, trnC-GCA, trnD-GUC, trnE-UUC, trnF-GAA, trnfM-CAU, trnG-GCC, trnG-UCC, trnH-GUG, trnI-GAU, trnK-UUU, trnL-CAA, trnL-UAA, trnL-UAG, trnM-CAU, trnN-GUU, trnP-UGG, trnQ-UUG, trnR-ACG, trnR-CCG, trnR-UCU, trnS-GCU, trnS-GGA, trnS-UGA, trnT-GGU, trnT-UGU, trnV-GAC, trnV-UAC, trnW-CCA, trnY-GUA* |
|  | Translational initiation factor | *infA* |
| Mitochondrion | ATP synthase subunits | *atp1, atp4, atp6, atp8, atp9* |
|  | Cytochrome c maturation genes | *ccmB, ccmC, ccmFC, ccmFN* |
|  | Cytochrome bc_1_ subunit | *cob* |
|  | Cytochrome c oxidase subunits | *cox1, cox2, cox3* |
|  | Large subunit of ribosome | *rpl2, rpl5, rpl6, rpl10, rpl16* |
|  | NADH dehydrogenase subunits | *nad1, nad2, nad3, nad4, nad4L, nad5, nad6, nad9* |
|  | Reverse transcriptase-like | *rtl* |
|  | Ribosomal RNA genes | *rrn5, rrn18, rrn26* |
|  | Small subunit of ribosome | *rps1, rps2, rps3, rps4, rps7, rps8, rps10, rps11, rps12, rps13, rps14, rps19* |
|  | Succinate dehydrogenase subunits | *sdh3, sdh4* |
|  | Transfer RNA genes | *trnA-UGC, trnC-GCA, trnD-GUC, trnE-UUC, trnF-GAA, trnG-GCC, trnG-UCC, trnH-GUG, trnI-CAU, trnK-UUU, trnL-CAA, trnL-UAA, trnL-UAG, trnM-CAU, trnMf-CAU, trnN-GUU, trnP-UGG, trnQ-UUG, trnR-ACG, trnR-UCU, trnS-GCU, trnS-UGA, trnV-UAC, trnW-CCA, trnY-GUA* |
|  | Twin arginine subunit c | *tatC* |
