# Supplementary figures and images for: Sequencing of organellar genomes of Gymnomitrion concinnatum (Jungermanniales) revealed the first exception in the structure and gene order of evolutionary stable liverworts mitogenomes
Source: BMC Plant Biol. 2018 Dec 3;18:321. doi: 10.1186/s12870-018-1558-0 (PMC6276189; doi:10.1186/s12870-018-1558-0)

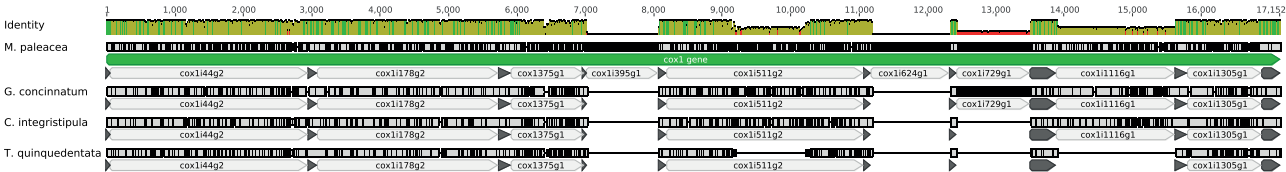

Supplement: Supplementary file 2 — Figure S1. Structure of cox1 gene among liverworts. The light grey coloured blocks depict introns while dark grey blocks depict exons of the gene. The M. paleacea cox1 gene is representative of the rest of liverworts. The consensus graph presents sequence identity among these four sequences (the greener regions the higher identity). (PDF 580 kb) [file 12870_2018_1558_MOESM2_ESM.pdf]
