# Supplementary material for: Sequencing of organellar genomes of Gymnomitrion concinnatum (Jungermanniales) revealed the first exception in the structure and gene order of evolutionary stable liverworts mitogenomes
Source: BMC Plant Biol. 2018 Dec 3;18:321. doi: 10.1186/s12870-018-1558-0 (PMC6276189; doi:10.1186/s12870-018-1558-0)
Supplement: Supplementary file 3 — Table S3. Predicted RNA editing sites within chloroplast genes of Gymnomitrion concinnatum. (DOC 149 kb) [file 12870_2018_1558_MOESM3_ESM.doc]

Table S3

Predicted RNA editing sites within chloroplast genes of *Gymnomitrion concinnatum*

| **Gene** | **C/U** | **U/C** | **Sum** | **Length**  **[bp]** | **Effect** | **ES Ratio**  **[%]** | **C/U Ratio**  **[%]** | **U/C Ratio**  **[%]** |
| --- | --- | --- | --- | --- | --- | --- | --- | --- |
| *accD* | 1 | 2 | 3 | 957 | 3 a. s. | 0.3135 | 0.1045 | 0.2090 |
| *atpA* | 5 | 2 | 7 | 1524 | 7 a. s. | 0.4593 | 0.3281 | 0.1312 |
| *atpB* | 3 | 2 | 5 | 1479 | 5 a. s. | 0.3381 | 0.2028 | 0.1352 |
| *atpE* | 2 | 0 | 2 | 417 | 2 a. s. | 0.4796 | 0.4796 | 0 |
| *atpF* | 4 | 1 | 5 | 555 | 4 a. s.  1start | 0.9009 | 0.7207 | 0.1802 |
| *atpH* | 1 | 0 | 1 | 246 | 1 a. s. | 0.4065 | 0.4065 | 0 |
| *atpI* | 4 | 0 | 4 | 723 | 4 a. s. | 0.5533 | 0.5533 | 0 |
| *ccsA* | 12 | 11 | 23 | 969 | 18 a. s.  1 stop | 2.3736 | 1.2384 | 1.1352 |
| *cemA* | 14 | 6 | 20 | 1344 | 19 a. s. | 1.4881 | 1.0417 | 0.4464 |
| *chlB* | 1 | 2 | 3 | 1542 | 3 a. s. | 0.1946 | 0.0649 | 0.1297 |
| *chlL* | 1 | 1 | 2 | 873 | 1 a. s. | 0.2291 | 0.1145 | 0.1145 |
| *chlN* | 5 | 0 | 5 | 1401 | 5 a. s. | 0.3569 | 0.3569 | 0 |
| *clpP* | 3 | 0 | 3 | 612 | 3 a. s. | 0.4902 | 0.4902 | 0 |
| *cysA* | 9 | 7 | 16 | 1086 | 15 a. s. | 1.4733 | 0.8287 | 0.6446 |
| *cysT* | 3 | 9 | 12 | 867 | 12 a. s. | 1.3841 | 0.3460 | 1.0381 |
| *infA* | 1 | 1 | 2 | 237 | 2 a. s. | 0.8439 | 0.4219 | 0.4219 |
| *matK* | 18 | 7 | 25 | 1074 | 24 a. s. | 2.3277 | 1.6760 | 0.6518 |
| *ndhA* | 10 | 5 | 15 | 1110 | 12 a. s. | 1.3514 | 0.9009 | 0.4505 |
| *ndhB* | 16 | 8 | 24 | 1503 | 24 a. s. | 1.5968 | 1.0645 | 0.5323 |
| *ndhC* | 5 | 3 | 8 | 363 | 7 a. s. | 2.2039 | 1.3774 | 0.8264 |
| *ndhD* | 11 | 9 | 20 | 1509 | 19 a. s. | 1.3254 | 0.7290 | 0.5964 |
| *ndhE* | 2 | 0 | 2 | 303 | 2 a. s. | 0.6601 | 0.6601 | 0 |
| *ndhF* | 33 | 13 | 46 | 2109 | 42 a. s. | 2.1811 | 1.5647 | 0.6164 |
| *ndhG* | 1 | 5 | 6 | 583 | 6 a. s. | 1.0292 | 0.1715 | 0.8576 |
| *ndhH* | 7 | 3 | 10 | 1182 | 10 a. s. | 0.8460 | 0.5922 | 0.2538 |
| *ndhI* | 2 | 3 | 5 | 555 | 5 a. s. | 0.9009 | 0.3604 | 0.5405 |
| *ndhJ* | 3 | 0 | 3 | 519 | 3 a. s. | 0.5780 | 0.5780 | 0 |
| *ndhK* | 3 | 6 | 9 | 747 | 8 a. s. | 1.2048 | 0.4016 | 0.8032 |
| *petA* | 7 | 6 | 13 | 963 | 12 a. s. | 1.3499 | 0.7269 | 0.6231 |
| *petB* | 3 | 0 | 3 | 648 | 3 a. s. | 0.4630 | 0.4630 | 0 |
| *petD* | 1 | 0 | 1 | 483 | 1 stop | 0.2070 | 0.2070 | 0 |
| *petG* | 0 | 0 | 0 | 114 | 0 | 0 | 0 | 0 |
| *petL* | 2 | 2 | 4 | 96 | 3 a. s. | 4.1667 | 2.0833 | 2.0833 |
| *petN* | 1 | 0 | 1 | 90 | 1 a. s. | 1.1111 | 1.1111 | 0 |
| *psaA* | 6 | 2 | 8 | 2253 | 7 a. s. | 0.3551 | 0.2663 | 0.0888 |
| *psaB* | 9 | 2 | 11 | 2205 | 11 a. s. | 0.4989 | 0.4082 | 0.0907 |
| *psaC* | 0 | 2 | 2 | 246 | 1 a. s. | 0.8130 | 0 | 0.8130 |
| *psaI* | 1 | 0 | 1 | 111 | 1 a. s. | 0.9009 | 0.9009 | 0 |
| *psaJ* | 2 | 0 | 2 | 129 | 2 a. s. | 1.5504 | 1.5504 | 0 |
| *psaM* | 2 | 0 | 2 | 99 | 2 a. s. | 2.0202 | 2.0202 | 0 |
| *psbA* | 0 | 0 | 0 | 1062 | 0 | 0 | 0 | 0 |
| *psbB* | 2 | 4 | 6 | 1518 | 5 a. s. | 0.3953 | 0.1318 | 0.2635 |
| *psbC* | 2 | 2 | 4 | 1422 | 4 a. s. | 0.2813 | 0.1406 | 0.1406 |
| *psbD* | 0 | 0 | 0 | 1062 | 0 | 0 | 0 | 0 |
| *psbE* | 0 | 0 | 0 | 252 | 0 | 0 | 0 | 0 |
| *psbF* | 0 | 1 | 1 | 120 | 1 a. s. | 0.8333 | 0 | 0.8333 |
| *psbH* | 1 | 0 | 1 | 225 | 1 a. s. | 0.4444 | 0.4444 | 0 |
| *psbI* | 0 | 0 | 0 | 120 | 0 | 0 | 0 | 0 |
| *psbJ* | 0 | 0 | 0 | 123 | 0 | 0 | 0 | 0 |
| *psbK* | 6 | 0 | 6 | 171 | 6 a. s. | 3.5088 | 3.5088 | 0 |
| *psbL* | 0 | 0 | 0 | 117 | 0 | 0 | 0 | 0 |
| *psbM* | 1 | 0 | 1 | 105 | 1 a. s. | 0.9524 | 0.9524 | 0 |
| *psbN* | 1 | 0 | 1 | 132 | 1 a. s. | 0.7576 | 0.7576 | 0 |
| *psbT* | 0 | 0 | 0 | 108 | 0 | 0 | 0 | 0 |
| *psbZ* | 2 | 0 | 2 | 189 | 2 a. s. | 1.0582 | 1.0582 | 0 |
| *rbcL* | 4 | 0 | 4 | 1428 | 4 a. s. | 0.2801 | 0.2801 | 0 |
| *rpl14* | 0 | 0 | 0 | 369 | 0 | 0 | 0 | 0 |
| *rpl16* | 0 | 0 | 0 | 426 | 0 | 0 | 0 | 0 |
| *rpl2* | 4 | 1 | 5 | 831 | 5 a. s. | 0.6017 | 0.4813 | 0.1203 |
| *rpl20* | 2 | 3 | 5 | 357 | 5 a. s. | 1.4006 | 0.5602 | 0.8403 |
| *rpl21* | 3 | 2 | 5 | 357 | 5 a. s. | 1.4006 | 0.8403 | 0.5602 |
| *rpl22* | 1 | 1 | 2 | 363 | 2 a. s. | 0.5510 | 0.2755 | 0.2755 |
| *rpl23* | 0 | 1 | 1 | 276 | 1 a. s. | 0.3623 | 0 | 0.3623 |
| *rpl32* | 2 | 0 | 2 | 201 | 2 a. s. | 0.9950 | 0.9950 | 0 |
| *rpl33* | 1 | 0 | 1 | 198 | 1 a. s. | 0.5051 | 0.5051 | 0 |
| *rpl36* | 0 | 0 | 0 | 114 | 0 | 0 | 0 | 0 |
| *rpoA* | 8 | 6 | 14 | 1011 | 12 a. s. | 1.3848 | 0.7913 | 0.5935 |
| *rpoB* | 12 | 4 | 16 | 3195 | 15 a. s. | 0.5008 | 0.3756 | 0.1252 |
| *rpoC1* | 10 | 8 | 18 | 2079 | 17 a. s. | 0.8658 | 0.4810 | 0.3848 |
| *rpoC2* | 36 | 34 | 70 | 4171 | 66 a. s. | 1.6783 | 0.8631 | 0.8152 |
| *rps11* | 1 | 0 | 1 | 393 | 1 a. s. | 0.2545 | 0.2545 | 0 |
| *rps12* | 0 | 0 | 0 | 258 | 0 | 0 | 0 | 0 |
| *rps14* | 2 | 1 | 3 | 303 | 3 a. s. | 0.9901 | 0.6601 | 0.3300 |
| *rps15* | 4 | 1 | 5 | 267 | 5 a. s. | 1.8727 | 1.4981 | 0.3745 |
| *rps18* | 0 | 0 | 0 | 228 | 0 | 0 | 0 | 0 |
| *rps19* | 0 | 1 | 1 | 279 | 1 a. s. | 0.3584 | 0 | 0.3584 |
| *rps2* | 4 | 1 | 5 | 714 | 5 a. s. | 0.7003 | 0.5602 | 0.1401 |
| *rps3* | 2 | 1 | 3 | 657 | 3 a. s. | 0.4566 | 0.3044 | 0.1522 |
| *rps4* | 6 | 7 | 13 | 609 | 11 a. s. | 2.1346 | 0.9852 | 1.1494 |
| *rps7* | 1 | 1 | 2 | 468 | 2 a. s. | 0.4274 | 0.2137 | 0.2137 |
| *rps8* | 0 | 1 | 1 | 399 | 1 a. s. | 0.2506 | 0 | 0.2506 |
| *ycf1* | 28 | 33 | 61 | 3156 | 56 a. s. | 1.9328 | 0.8872 | 1.0456 |
| *ycf12* | 0 | 1 | 1 | 102 | 1 a. s. | 0.9804 | 0 | 0.9804 |
| *ycf2* | 71 | 56 | 127 | 6117 | 115 a. s. | 2.0762 | 1.1607 | 0.9155 |
| *ycf3* | 0 | 2 | 2 | 504 | 1 a. s. | 0.3968 | 0 | 0.3968 |
| *ycf4* | 6 | 1 | 7 | 555 | 7 a. s. | 1.2613 | 1.0811 | 0.1802 |
| *ycf66* | 7 | 1 | 8 | 442 | 7 a. s. | 1.8100 | 1.5837 | 0.2262 |

The 'C/U' and 'U/C' columns depict number of C to U and U to C RNA substitutions within protein-coding sequences. The 'Sum' depicts total number of identified RNA editing substitutions. The 'Length' column represents the length of CDS of the genes. The effects of RNA editing substitutions are shown in 'Effect' column, where 'start' means substitution that cause occurrence of translation initiation codon, 'stop' - substitution that cause occurrence of translation termination codon and 'a. s.' - substitution that cause occurrence of alternative amino acid codon without translation initiation nor termination effect. The last three columns depict the percent of: total number ('ES Ratio'), number of C to U ('C/U Ratio') and number of U to C ('U/C Ratio') RNA editing substitutions per CDS length.
