# Supplementary material for: Sequencing of organellar genomes of Gymnomitrion concinnatum (Jungermanniales) revealed the first exception in the structure and gene order of evolutionary stable liverworts mitogenomes
Source: BMC Plant Biol. 2018 Dec 3;18:321. doi: 10.1186/s12870-018-1558-0 (PMC6276189; doi:10.1186/s12870-018-1558-0)

LCBs pair A-D: expected amplicon size - 1797 bp; obtained - 1799 bp (±10 bp)

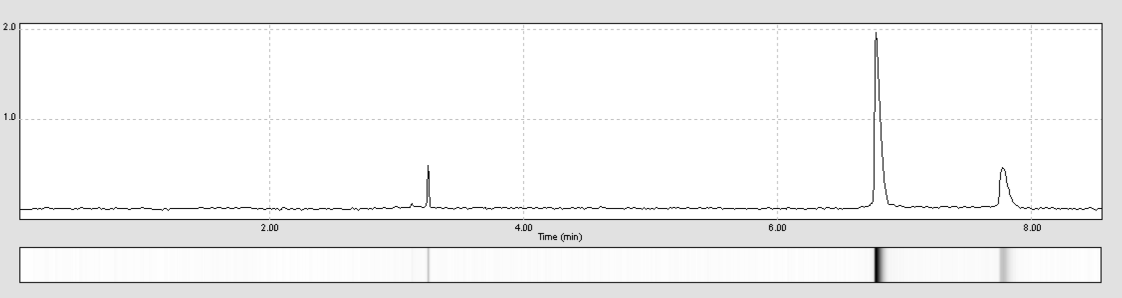

LCBs pair D-B: expected amplicon size - 1605 bp; obtained -1608 bp (±10 bp)

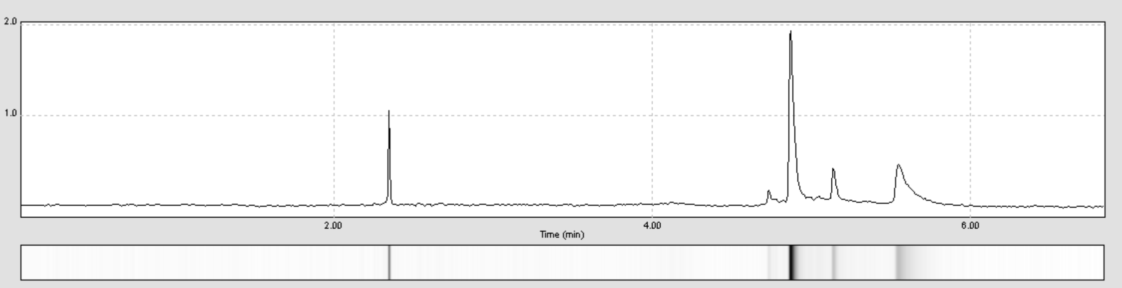

LCBs pair B-C: expected amplicon size - 1810 bp; obtained - 1813 bp (±10 bp)

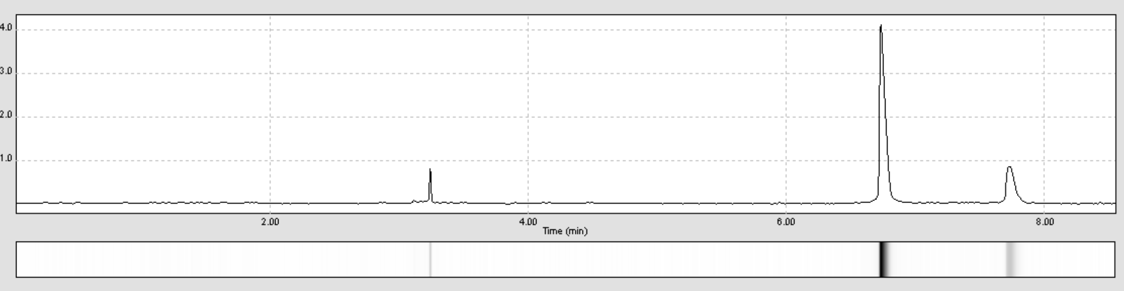

LCBs pair C-E: expected amplicon size - 1602 bp; obtained - 1610 bp (±10 bp)

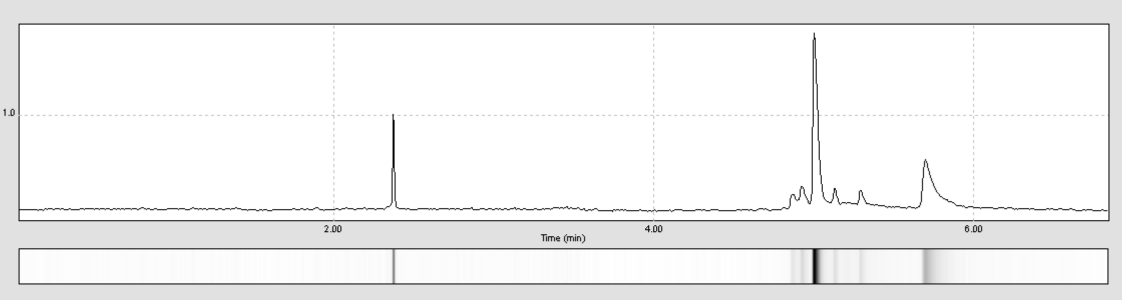

Supplement: Supplementary file 5 — Figure S2. The PCR validation of the mitogenome structure. Four electropherograms of amplicons obtained as a result of PCR analysis. The x-axis represents time while y-axis represents relative fluorescence. (PDF 209 kb) [file 12870_2018_1558_MOESM5_ESM.pdf]
