# Supplementary material for: Sequencing of organellar genomes of Gymnomitrion concinnatum (Jungermanniales) revealed the first exception in the structure and gene order of evolutionary stable liverworts mitogenomes
Source: BMC Plant Biol. 2018 Dec 3;18:321. doi: 10.1186/s12870-018-1558-0 (PMC6276189; doi:10.1186/s12870-018-1558-0)
Supplement: Supplementary file 6 — Table S1. Sequences of primers used in the present study. (DOC 37 kb) [file 12870_2018_1558_MOESM6_ESM.doc]

Table S1

Sequences of primers used in the present study

| **LCB**  **pair** | **Direction** | **Primer sequence (5’- 3’)** | **Excepted amplicon size [bp]** |
| --- | --- | --- | --- |
| A-D | forward | CTGCCATGGCCCAAAACAAA | 1797 |
|  | reverse | TCGGCCACACACGTTTATGA |  |
| D-B | forward | ACAAGGGAAGGGGCTTAAGC | 1605 |
|  | reverse | TGTACGATACGCGGGCTAAC |  |
| B-C | forward | TTGGATTGGCTACTCCTGCG | 1810 |
|  | reverse | CTGTCATTGTAGCCGGGGTT |  |
| C-E | forward | TAGCCTACCCTGCAGTGGAT | 1602 |
|  | reverse | ATATGTGCTCAAGGCCTCCG |  |
